# The Concept of Anatomical Reconstruction of the Foveola Using Activated Conditioned Plasma (ACP)

**DOI:** 10.3390/jcm14155358

**Published:** 2025-07-29

**Authors:** Monika Popowska, Ludmila Popowska, Leonid I. Balashevich, Jacek P. Szaflik, Monika Łazicka-Gałecka

**Affiliations:** 1 SPKSO Ophthalmic University Hospital Warsaw, 03-709 Warsaw, Poland; jacek.szaflik@wum.edu.pl (J.P.S.); lazgal@wp.pl (M.Ł.-G.); 2 LUMED Clinic, 26-300 Opoczno, Poland; poplud@interia.pl; 3 St. Petersburg Branch of the S. Fyodorov Eye Microsurgery Federal State Institution, 127486 Moscow, Russia; 4 Laser Eye Microsurgery Centre in Warsaw, 00-215 Warszawa, Poland; 5 Department of Ophthalmology, Medical University of Warsaw, 02-091 Warszawa, Poland

**Keywords:** macular hole, internal limiting membrane (ILM), activated conditioned plasma (ACP), intraoperative OCT, fibrin membrane, primary tension closure, best corrected visual acuity (BCVA)

## Abstract

**Background:** Surgical management of large full-thickness macular holes (MHs) remains challenging, particularly when aiming for both rapid visual recovery and consistent anatomical closure without inducing retinal trauma. This retrospective single-center study evaluated the efficacy of activated conditioned plasma (ACP) as an intraoperative coadjuvant supporting ILM (internal limiting membrane) peeling and air tamponade in the treatment of idiopathic MHs measuring 400–800 µm, under real-time intraoperative optical coherence tomography (i-OCT) guidance. **Methods:** Seventy eyes from fifty patients underwent pars plana vitrectomy with intraoperative ACP application. ACP, a leukocyte-poor autologous platelet concentrate, was used intraoperatively as a coadjuvant to ILM peeling and air tamponade. It facilitated the formation of a transparent fibrin membrane over the retinal surface, supporting edge approximation and promoting retinal healing. **Results:** The primary outcome was complete MH closure confirmed by OCT; the secondary outcome was improvement in BCVA on postoperative day 7 and during a 12-month follow-up. Anatomical closure was achieved in 98.6% of cases. On day 7, 78.6% of eyes showed a ≥ three-line BCVA improvement, with mean BCVA increasing from 0.25 ± 0.21 to 0.69 ± 0.20 (*p* < 0.001). These outcomes remained stable throughout the follow-up. No significant intraoperative or postoperative complications were observed. **Conclusions:** The combination of ACP and i-OCT appears to be a safe and effective strategy for anatomical foveolar reconstruction, enabling early visual recovery while minimizing inflammation and fibrotic scarring associated with conventional techniques.

## 1. Background

Full-thickness macular holes (MHs) are a significant cause of central vision impairment, with a prevalence ranging from 0.2% to 0.8% in the general population, affecting predominantly women and individuals over the age of 60 [1,2]. Despite considerable advances in vitreoretinal surgery, the management of large MHs (greater than 400 µm) remains a challenge, with suboptimal visual recovery even in anatomically successful cases.

The term “macular hole” has long been used in ophthalmic nomenclature, yet it may oversimplify the true pathomorphological nature of the condition. The word “hole” implies an empty space to be filled or plugged. In contrast, current understanding suggests that full-thickness MHs should be regarded as mechanical retinal ruptures, involving tissue discontinuity rather than simple absence of structure [3,4]. This conceptual shift carries important clinical implications, promoting a surgical strategy focused on active approximation and biological stabilization of retinal tissue, rather than passive sealing with exogenous materials.

Several mechanisms have been implicated in the formation of macular ruptures, including horizontal vitreoretinal traction (particularly tangential forces at the foveola), sudden posterior vitreous detachment (PVD), structural changes and fibrosis of the ILM, and secondary causes such as trauma, high myopia, diabetes, or previous ocular procedures [4,5,6].

Conventional surgical techniques typically involve pars plana vitrectomy (PPV) with ILM peeling and gas or silicone oil tamponade. More advanced methods include the inverted ILM flap technique or the use of biological adjuvants such as PRP, amniotic membrane, or collagen matrix [7,8,9]. Although these approaches have demonstrated high closure rates, they often leave non-physiological material on the retinal surface, potentially promoting gliotic scar formation and limiting neuroretinal recovery [10,11]. Moreover, PRP’s (platelet-rich plasma) high leukocyte and erythrocyte content has been linked to increased postoperative inflammation and delayed wound healing [11,12].

The present study introduces an alternative concept based on the use of ACP, a leukocyte-poor concentrate capable of forming a transparent fibrin membrane in situ. This membrane functions as a temporary biological scaffold that facilitates edge approximation and supports neuroepithelial regeneration without leaving foreign tissue or inducing chronic inflammation. Intraoperative optical coherence tomography (i-OCT) enables real-time visualization of the macular hole margins, allowing for precise control of membrane placement and tissue positioning [3,4,10].

Therefore, the aim of this study is to evaluate the safety and efficacy of ACP in the treatment of large idiopathic macular holes (400–800 µm), with particular attention to early postoperative visual outcomes, surgical control, and the avoidance of pro-inflammatory sequelae associated with traditional techniques.

## 2. Materials and Methods

### 2.1. Study Design

This retrospective, single-center study included 50 patients (70 eyes) who underwent pars plana vitrectomy (PPV) combined with ACP application for the treatment of idiopathic full-thickness MHs between 2018 and 2024. The study adhered to the principles of the Declaration of Helsinki and received approval from the Local Bioethics Committee (approval number: AKBE/27/2025). Informed consent was obtained from all participants.

### 2.2. Statistical Methods

The paired Student’s *t*-test was applied to compare mean BCVA values measured one day before surgery, on postoperative day 7, and at the 12-month follow-up. A *p*-value < 0.001 was considered statistically significant based on the results obtained.

### 2.3. Inclusion and Exclusion Criteria

The inclusion criteria were as follows:

(1) Idiopathic full-thickness macular holes;
(2) Minimum linear diameter of the hole between 400 and 800 µm;
(3) Age ≥ 18 years;
(4) Complete preoperative and postoperative data available;
(5) Informed consent for surgery and participation.

The exclusion criteria were as follows:

(1) Secondary MHs due to trauma, high myopia, or retinal vascular diseases;
(2) Previous retinal surgery in the studied eye;
(3) Concomitant advanced glaucoma or uveitis;
(4) Retinal detachment.

### 2.4. Preoperative and Postoperative Assessment

All patients underwent a comprehensive ophthalmological examination including BCVA, intraocular pressure (IOP) measurement, slit-lamp biomicroscopy, dilated fundus examination, and spectral-domain optical coherence tomography (OCT). BCVA was measured using a decimal Snellen chart at a distance of 6 m under standardized ambient photopic conditions. Values were recorded in decimal format and converted into logMAR units for statistical evaluation. All measurements were performed by trained ophthalmic personnel under consistent clinical settings.

Follow-up visits were scheduled at day 1, day 7, 1 month, 3 months, 6 months, and 12 months postoperatively.

The primary outcome was defined as complete anatomical closure of the macular hole confirmed by OCT. The secondary outcome was defined as improvement in BCVA recorded on day 7 and at subsequent follow-up visits.

Intraoperative and postoperative complications such as retinal tears, elevated IOP, vitreous hemorrhage, fibrin reaction, or reopening of the MH were systematically documented.

### 2.5. Surgical Technique

A subtotal pars plana vitrectomy was performed using the CONSTELLATION^®^ Vision System (Alcon, Fort Worth, TX, USA). The posterior hyaloid membrane was visualized with a suspension of triamcinolone acetonide and gently detached using a ULTRAVIT^®^ 27+ vitreotome (Alcon, Fort Worth, TX, USA).

**Figure 1:**
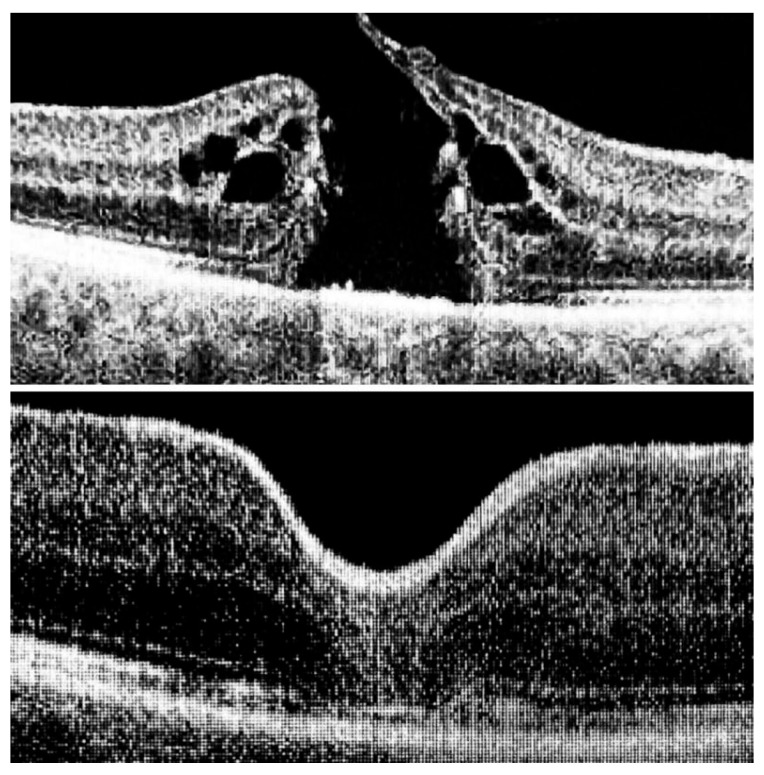
Intraoperative images before ILM peeling and after macular hole closure, with superficial approximation of the edges using a silicone-tipped cannula.

**Figure 2:**
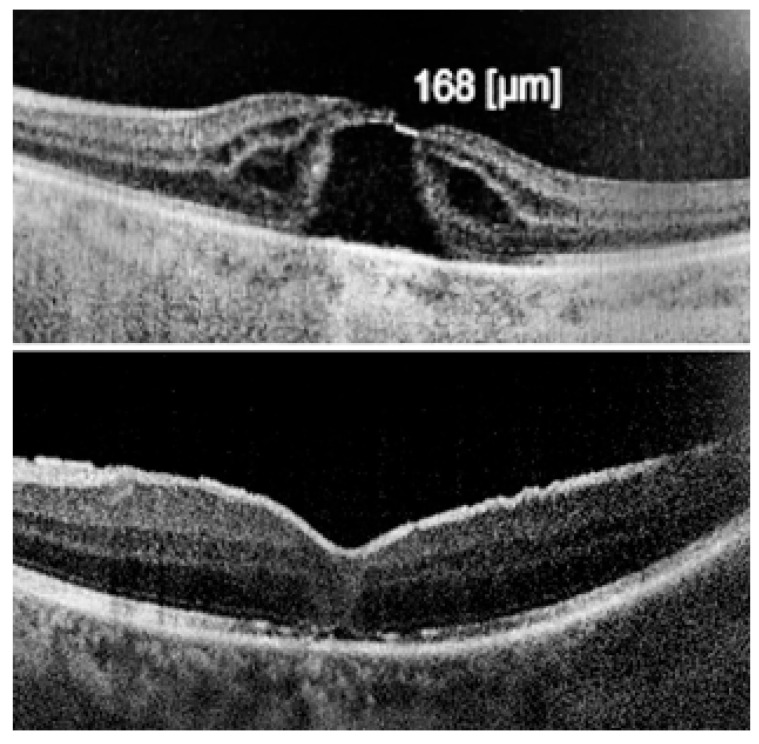
Top image: visible internal limiting membrane (ILM); bottom image: ILM removed—without ILM peeling, perfect macular hole closure is not achievable.

**Figure 3:**
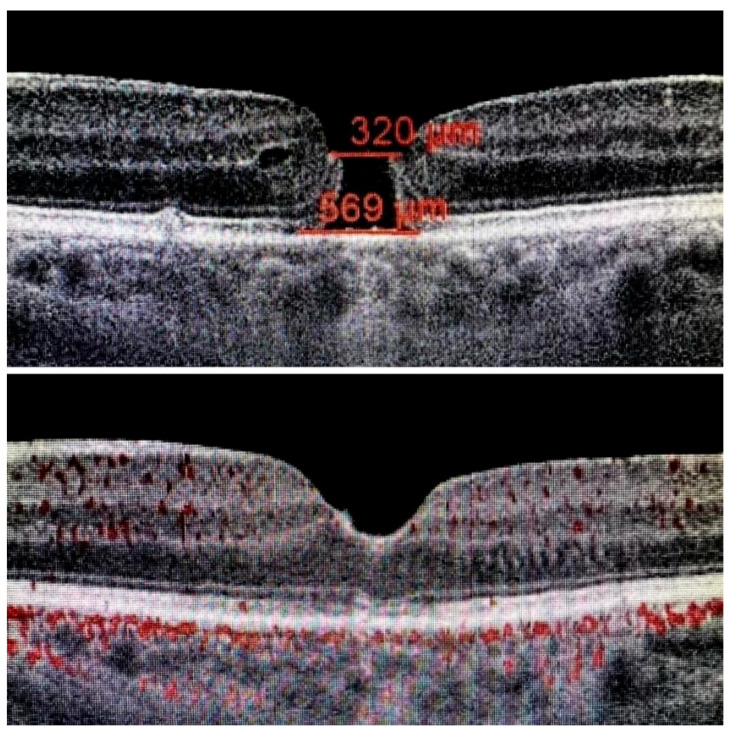
At the top: long-standing macular hole persisting for 12 months; at the bottom: complete closure with restoration of all retinal layers, preserving ideal architecture and morphology, including the ninth layer—the retinal pigment epithelium (RPE).

The ILM was peeled without the use of vital dyes, employing visualization techniques for transparent structures. A 25G silicone soft-tip cannula was used to gently approximate the edges of the macular hole under a balanced salt solution. Triamcinolone aceonide (Kenalog) was deposited on the ILM surface to enhance its visibility, and additional guidance was provided by instrument shadows under green-light illumination. Peeling was initiated at the point of weakest adhesion between the ILM and the retina. A pair of vitreoretinal forceps (e.g., Alcon Grieshaber AG, Schaffhausen, Switzerland) was used to gently grasp and lift the ILM to initiate separation. The extent of peeling covered an area of approximately 2–3 optic disc diameters around the hole to relieve tangential traction. Following ILM peeling and centripetal tissue mobilization, the macular hole edges were approximated under balanced salt solution or, in selected cases, perfluorocarbon liquid. Closure was monitored in real time using high-resolution intraoperative OCT (RESCAN™ 700 integrated with OPMI LUMERA^®^ 700 microscope, Carl Zeiss Meditec AG, Jena, Germany). Representative intraoperative OCT images before and after ACP application are presented in Figure 1, Figure 2 and Figure 3.

ACP was applied over the retinal surface in the area of ILM removal. To ensure safe and controlled delivery, ACP was aspirated into two sterile 1 mL syringes. Although the total volume prepared was 1–2 mL, only a single drop (approximately 0.02 mL) was applied over the macular hole under i-OCT guidance.

Within 2–3 min, i-OCT confirmed the formation of a transparent fibrin membrane tightly adherent to the exposed retina, effectively sealing the macular hole and preventing fluid ingress. At the end of surgery, the intraocular fluid was exchanged with air. Patients were instructed to maintain a face-down position for 10 h postoperatively.

After this initial period, patients were allowed to assume any comfortable posture, such as sitting or lying on the side, while strictly avoiding the supine position. This recommendation was intended to prolong macular exposure to the air tamponade and to minimize the risk of lens opacification in phakic eyes. The recommendation for early face-down positioning following surgery is supported by findings from a Cochrane systematic review, which evaluated the impact of postoperative posturing on macular hole closure rates [13].

All procedures were performed by a single experienced vitreoretinal surgeon under general anesthesia (TIVA).

### 2.6. ACP Preparation

ACP is an autologous, leukocyte-poor platelet concentrate, commonly used in regenerative medicine and recently adapted for intraocular applications. The details of ACP preparation using the Arthrex system are presented in Table 1. In this study, ACP was prepared intraoperatively using the Arthrex ACP Double Syringe System. A total of 15 mL of autologous venous blood were collected during surgery, followed by centrifugation at 1600 rpm for 5 min. This process yielded 5 mL of leukocyte-poor plasma. No additional processing or exogenous activation was applied prior to intraocular use.

Routine sterility testing was not performed, as the autologous origin of ACP, the preoperative hematologic screening of all patients, and the low leukocyte content significantly minimized the risk of contamination or immune response. Furthermore, no cases of intraocular infection or inflammation were observed in the postoperative period.

The resulting ACP contains a moderate concentration of platelets and a significantly reduced leukocyte and erythrocyte content compared to classical PRP preparations. This profile minimizes the risk of pro-inflammatory reactions and supports tissue regeneration.

Representative values based on intraoperative ACP preparation using the Arthrex system, consistent with findings reported by Fitzpatrick et al. [9].

## 3. Results

Visual acuity improved significantly in the majority of cases. The mean preoperati-ve BCVA was 0.25 ± 0.21. Baseline demographic and clinical characteristics of the study group are summarized in Table 2. On postoperative day 7, the mean BCVA improved to 0.69 ± 0.20 (*p* < 0.001; paired Student’s *t*-test). Notably, 55 eyes (78.6%) achieved an improvement of at least three lines on the Snellen chart by day 7 postoperatively. A detailed summary of visual acuity outcomes is presented in Table 3. Macular hole size ranged from 400 to 800 µm. The mean diameter was 448 µm (SD: 52 µm). Only two cases exceeded 700 µm. The duration of disease ranged from 0.5 to 14 months, with a mean of 4.2 months. All patients completed the 12-month postoperative follow-up.

Cataract progression was observed as a common postoperative event. In the studied cohort, 59 out of 70 eyes (84.3%) developed cataracts within six months following vitrectomy. Table 4 presents postoperative outcomes at 12-month follow-up, including cataract progression and anatomical closure status.

## 4. Discussion

The surgical management of large full-thickness MHs remains a significant challenge in vitreoretinal surgery. This study introduces a conceptually novel approach, viewing MHs as mechanical ruptures of retinal tissue rather than empty defects. This paradigm shift has implications for surgical technique, emphasizing precise edge approximation and biologically integrated closure.

Our results demonstrate that the use of ACP, combined with intraoperative optical coherence tomography (i-OCT), is associated with a high closure rate of 98.6% and early functional improvement, with 78.6% of patients gaining three or more Snellen lines of BCVA by day 7. These outcomes compare favorably to those achieved using techniques such as internal limiting membrane (ILM) peeling with gas tamponade or inverted ILM flap procedures.

The in situ formation of a transparent fibrin membrane following ACP application provides temporary stabilization of the macular rupture and serves as a scaffold for neuroepithelial regeneration. The membrane is fully reabsorbed, leaving no residual material. I-OCT allowed real-time visualization and control of macular hole edge approximation and membrane application, which likely contributed to the favorable anatomical and functional results.

**Figure 4:**
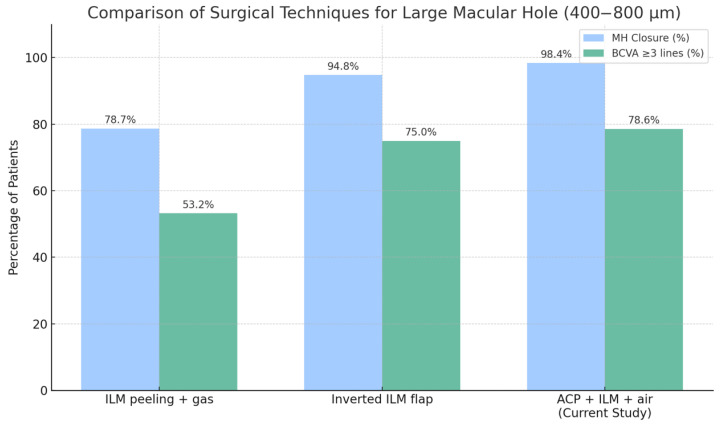
Comparison of the efficacy of different surgical techniques for the treatment of large full-thickness macular holes (400–800 µm). Blue bars represent the MH closure rate, and green bars indicate the percentage of patients with a ≥ 3-line improvement in BCVA. Data for ILM peeling and inverted ILM flap techniques were obtained from the meta-analysis by Chen et al. [14] (2020, *PLoS ONE*), while data for ACP + ILM peeling + air tamponade originate from the present study.

**Figure 5:**
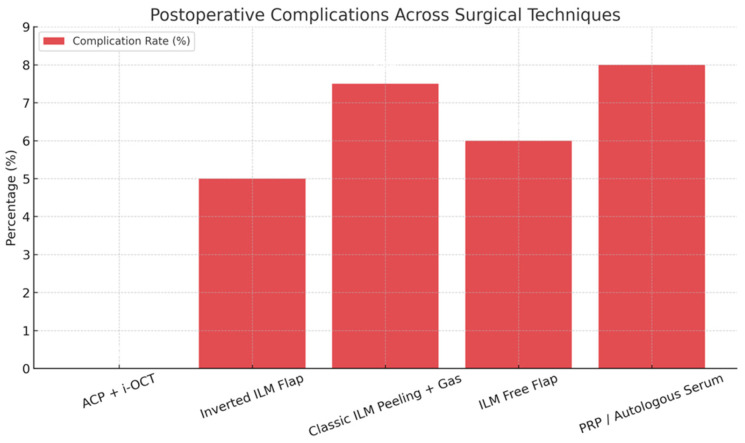
Comparison of postoperative complications reported in the literature for different surgical techniques used in the treatment of large full-thickness macular holes. Complications included the following: transient intraocular pressure elevation [14], inflammatory (fibrinous) reaction [11], retinal pigment epithelium (RPE) atrophy [4,5], and retinal phototoxicity [6]. No complications were observed in the ACP + ILM peeling + air tamponade group.

In two cases, a reduction in best corrected visual acuity of three or more Snellen lines was observed at both day 7 and 12-month follow-up. In both instances, retinal pigment epithelium (RPE) dysfunction was identified as the primary limiting factor. No intraoperative complications or reopenings were reported. A comparison of surgical outcomes and complications using different techniques is shown in Figure 4 and Figure 5.

### 4.1. Comparison with Platelet-Rich Plasma (PRP)

Several studies have investigated the use of autologous blood derivatives such as PRP in macular hole surgery. PRP contains a high concentration of leukocytes, erythrocytes, and inflammatory mediators, which may promote gliosis and fibrotic healing, potentially limiting functional outcomes [9,11,12]. ACP, in contrast, is a leukocyte-poor autologous plasma concentrate designed to minimize the risk of postoperative inflammation. It forms a transparent fibrin membrane that supports mechanical closure without inducing secondary tissue proliferation. The present study found that ACP reduced leukocyte content approximately ninefold compared to standard PRP preparations, which may contribute to its favorable clinical profile [9,11]. Earlier reports, including those by Liggett et al. [15] and Gotzaridis et al. [16], described anatomical closure with PRP or autologous serum, but without consistent visual improvement. The reduced inflammatory profile of ACP may address this limitation, suggesting a more suitable biological environment for macular healing [9,11,12].

### 4.2. Comparison with ILM Grafts

ILM grafting is a widely used technique for treating large MHs, providing mechanical coverage of the defect and improving anatomical closure rates. However, this approach often results in the formation of gliotic scar tissue within the foveal region, which may impair functional outcomes due to architectural distortion and limited neuroretinal recovery [17]. Morizane et al. reported anatomical closure rates of 85–90% using autologous ILM transplantation, yet emphasized that visual gains were frequently suboptimal, primarily due to glial proliferation within the grafted area [17].

In contrast, the ACP-based technique evaluated in the present study facilitates closure without introducing permanent or fibrotic material into the foveal space. The fibrin membrane formed by ACP is fully resorbable and supports tension-free sealing, minimizing the risk of gliosis and promoting more physiological neuroepithelial regeneration [10,11,18]. Additionally, i-OCT guidance ensures real-time monitoring of edge approximation and precise placement of the fibrin matrix, further enhancing surgical predictability and minimizing postoperative distortion [3,4].

These findings suggest that ACP combined with i-OCT may offer a superior alternative to ILM grafting for large MHs, particularly in cases where preservation of the foveal microarchitecture and early functional recovery are primary goals. Other biological adjuvants, such as human amniotic membrane plugs, have also been investigated in the treatment of complex or recurrent macular holes, offering an alternative scaffold for retinal repair [19].

### 4.3. Comparison with Gas Tamponade

Gas tamponade with face-down positioning is considered a standard approach for small and medium-sized MHs. However, its efficacy is reduced in large defects (>400 µm), where closure often relies on secondary connective tissue formation [20]. Altaweel et al. (2003) reported limited functional improvement when gas tamponade was used in large MHs [21]. In contrast, ACP promotes fibrin membrane formation, facilitating immediate macular hole edge approximation in a fluid-filled eye. The combination of ACP with i-OCT enables direct visualization and real-time adjustment of the MH edges, improving closure rates and limiting reliance on postoperative positioning or scar tissue formation [4,14,18].

### 4.4. Advantages of the ACP + Intra-OCT Approach

The combination of ACP and i-OCT offers several advantages over traditional methods. It is important to note that ACP was used exclusively as an intraoperative coadjuvant. All patients underwent standard surgical procedures including ILM peeling, mechanical edge approximation, and air tamponade. ACP enables rapid fibrin membrane formation with no inflammatory response due to its leukocyte-poor composition [4,11,18]. The i-OCT guidance ensures safe and precise edge approximation without the use of vital dyes, reducing the risk of phototoxicity and mechanical damage [3,10]. This strategy results in both a high closure rate and rapid functional recovery. The early BCVA improvement observed in this study, with stabilization over 12 months, supports the regenerative potential of this technique and its clinical applicability, as demonstrated by comparison with outcomes from recent meta-analyses, including Mihalache et al. (2024) [20]. A comparative summary of published outcomes is presented in Table 5.

### 4.5. Mechanism of Action of Activated Conditioned Plasma (ACP)

Unlike silicone oil or inverted ILM flaps, ACP forms a fully resorbable, in situ polymerized fibrin membrane that provides effective sealing without requiring removal or manipulation. It eliminates the need for vital dyes and prolonged tamponade, reducing risks of toxicity and mechanical trauma. The use of green-filtered illumination during surgery further protects the retinal pigment epithelium and ganglion cells, contributing to overall surgical safety. Activated conditioned plasma (ACP) is an autologous, leukocyte-poor platelet concentrate used in ophthalmology to stabilize macular hole edges and promote retinal tissue regeneration. Its mechanism of action involves three key biological processes:

Formation of a fibrin membrane.

Following intraocular application, especially in areas where the internal limiting membrane (ILM) has been removed, ACP undergoes rapid fibrin polymerization. Within approximately 5 min, fibrin monomers are converted into a fibrin polymer, resulting in the formation of a thin, transparent membrane firmly adherent to the retinal surface. This fibrin membrane acts as a biological scaffold that supports macular hole edge approximation and facilitates neuroepithelial regeneration.

Intraoperative OCT imaging confirmed the presence and stable adhesion of the fibrin membrane throughout the surgical procedure.

Prevention of subretinal fluid ingress.

Due to its favorable surface tension properties, the fibrin membrane effectively seals the macular hole, preventing subretinal fluid ingress. This mechanical barrier plays a crucial role in stabilizing the postoperative architecture and reducing the risk of hole reopening.

Activation of regenerative processes.

ACP promotes tissue regeneration through the localized release of platelet-derived growth factors, which are essential for wound healing and cellular proliferation. The reduced leukocyte concentration (WBC = 1.3 × 10^9^/L) minimizes postoperative inflammation, contributing to favorable anatomical and functional outcomes.

### 4.6. Study Limitations and Future Directions

Despite the encouraging results, this study has certain limitations. It was a retrospective, single-center investigation without a randomized control group, which may limit the strength of statistical inference. The sample size (70 eyes, 50 patients) was moderate, and the inclusion of both eyes from some individuals may have introduced intra-subject correlation that was not accounted for in the analysis. Although the follow-up period extended to 12 months, further studies are required to fully assess the durability of the structural and functional outcomes. It is worth emphasizing that the surgical procedure was highly standardized and guided by i-OCT, and the results were contextualized with data from recent meta-analyses on the treatment of large macular holes [14,20]. This comparative approach allows the findings to be positioned within a broader clinical and scientific framework. Given the very high anatomical closure rate and the early, sustained improvement in visual acuity, the presented technique merits further investigation. Future prospective, multicenter studies involving larger patient populations and direct comparison with established methods such as the inverted ILM flap or autologous PRP are warranted to validate both its anatomical and functional efficacy and define its role in routine clinical practice. Although Kenalog^®^ (triamcinolone acetonide Bristol-Myers Squibb, New York, NY, USA) was used intraoperatively to assist visualization of the posterior hyaloid and ILM, it should be noted that this formulation contains preservatives and is not specifically approved for intraocular use. While previous reports have highlighted potential concerns regarding off-label intravitreal administration, no adverse reactions or inflammatory complications were observed in our study cohort.

## 5. Conclusions

Activated conditioned plasma (ACP), applied under intraoperative OCT guidance, appears to be a safe and effective technique for managing large full-thickness macular holes (400–800 µm) [3,7,10]. The method yielded a 98.6% closure rate and early visual improvement in most patients without requiring gas tamponade, dyes, or grafts [9,11,14]. The fibrin membrane formed in situ promoted neuroretinal healing without adverse inflammatory responses [10,11,12]. These results support ACP as a promising alternative to conventional approaches. Further prospective studies are needed to validate and refine this technique [14,20].

## Figures and Tables

**Table 1:** A comprehensive analysis of plasma fraction is presented in the table below.

System	Platelets (×10^9^/L)	WBC (×10^9^/L)	RBC (×10^12^/L)
Control (whole blood)	269	8.75	4.7
ACP	412	1.3	0.08

**Table 2:** Demographic and clinical characteristics of patients.

Characteristic	Main Group
Macular hole size (µm)	400–800
Number of eyes	70
Number of patients	50
Gender	Women: 31 (62%)
	Men: 19 (38%)
Mean age (years)	61 ± 7.8 years (mean ± SD)
Mean age (years mean ± SD)	61 ± 7.2
BCVA before surgery (mean ± SD)	0.25 ± 0.21
Laterality of the operated eye	right: 38 (54%)
	left: 32 (46%)
Mean duration of symptoms	9.6 weeks (range: 2–24 weeks)

**Table 3:** Anatomical and functional outcomes in the study group on day 7 after surgery.

Patients Eyes = 70	Average BCVA (±SD)	Improvement ≥ 3 Lines (%)	No Change (%)	Deterioration ≥ 3 Lines (%)	Complete Closure of Macular Hole (%)
Study group eyes = 70	0.65 (±0.20)	55 (78.6%)	13 (18.6%)	2 (2.9%)	69 (98.6%)

**Table 4:** Postoperative outcomes at 12-month follow-up. The table illustrates the percentage of patients achieving significant visual improvement (≥3 Snellen lines), those with no change, and those experiencing visual deterioration. The anatomical closure rate was 98.4%.

Patients Eyes = 70	Average BCVA (±SD)	No Change *N* (%)	Improvement by ≥ 3 Lines *n* (%)	Deterioration ≥ 3 Lines (%)	RPE Atrophy *n* (%)	Complete Closure of Macular Hole *n* (%)
Study group (eyes = 70)	0.65 (±0.20)	14 (19.8%)	51 (73.5%)	2 (2.9%)	3 (4.5%)	69 (98.6%)

**Table 5:** Comparison of surgical techniques for large full-thickness macular holes (400–800 µm). Anatomical and functional outcomes based on data from the present study, Chen et al. [14], and Mihalache et al. [20].

Technique	Closure Rate (%)	≥3-Line BCVA Gain (%)	Source
ILM Peeling (classic)	85.0	63.0	Chen et al., 2020
Inverted ILM Flap	92.0	72.0	Chen et al., 2020
No ILM Peel	78.0	55.0	Mihalache et al., 2024
ACP + ILM Peel + i-OCT (this study)	98.6	78.6	Present study

## Data Availability

The data presented in this study are available on reasonable request from the corresponding author. Restrictions apply to the availability of these data due to patient privacy protection.
